# 30-day mortality prediction in acute upper gastrointestinal bleeding: Incremental value of the prognostic nutritional index with ABC and Rockall scores

**DOI:** 10.17305/bb.2026.13995

**Published:** 2026-03-09

**Authors:** Murat Das, Ozge Kurtkulagi, Ece Unal Cetin, Hakan Kayan, Senem Guler, Ferhan Demirer Aydemir, Feyza Mutlay, Adil Ugur Çetİn, Yavuz Beyazit

**Affiliations:** 1 Department of Emergency Medicine, Çanakkale Onsekiz Mart University, Çanakkale, Türkiye; 2 Department of Internal Medicine, Çanakkale Onsekiz Mart University, Çanakkale, Türkiye; 3 Department of Intensive Care, Çanakkale Onsekiz Mart University, Çanakkale, Türkiye; 4 Department of Geriatrics, Çanakkale Onsekiz Mart University, Çanakkale, Türkiye; 5 Department of Internal Medicine, Çanakkale Mehmet Akif Ersoy State Hospital, Çanakkale, Türkiye

**Keywords:** Upper gastrointestinal bleeding, prognostic nutritional index, mortality, Rockall score, ABC score, lactate

## Abstract

Mortality risk among patients admitted to the emergency department (ED) with acute upper gastrointestinal (GI) bleeding is heterogeneous, underscoring the importance of early identification of high-risk individuals. This study aimed to evaluate the prognostic performance of the prognostic nutritional index (PNI) in predicting 30-day mortality and to determine whether incorporating PNI into established risk markers enhances prognostic accuracy. In this retrospective cohort study, we analyzed data from 619 patients with acute upper GI bleeding who presented to a tertiary university hospital between January 1, 2018, and December 31, 2024. Demographic, clinical, and laboratory data were extracted from medical records. PNI was calculated using serum albumin and lymphocyte count at the time of admission, with the primary outcome being 30-day mortality. Predictors of mortality were examined using univariable and multivariable logistic regression analyses. The incremental prognostic value of PNI was evaluated through receiver operating characteristic (ROC) analysis and the DeLong test. The median age of participants was 74.0 years (interquartile range: 63.0–81.0), and 38% of the patients were female. The observed 30-day mortality rate was 7.9%. Non-survivors displayed significantly lower PNI levels compared to survivors (37.6 vs. 43.6; *P <* 0.001). In multivariable analysis, PNI (odds ratio [OR]: 0.847 [0.765–0.938]), lactate level (OR: 1.225 [1.047–1.434]), and the ABC score (OR: 1.201 [1.053–1.370]) were identified as independent predictors of mortality. The risk of mortality increased substantially when low PNI was combined with a high ABC score or elevated lactate level. Incorporating PNI into a baseline model resulted in a modest increase in the area under the receiver operating characteristic curve (AUROC) from 0.708 to 0.774 (*P* ═ 0.049). In conclusion, PNI serves as an independent predictor of 30-day mortality in patients with acute upper GI bleeding. Its integration with existing risk scores may enhance prognostic discrimination and facilitate early risk stratification in the ED.

## Introduction

Acute upper gastrointestinal bleeding (UGIB) is a common cause of emergency department (ED) admissions and is associated with significant morbidity and mortality, particularly among elderly patients and those with comorbidities [1]. Despite advancements in diagnostic and therapeutic approaches, mortality related to UGIB remains clinically significant, underscoring the importance of early identification of high-risk patients [2]. Consequently, there is a continued need for rapid, reliable, and user-friendly risk stratification tools at the time of ED admission.

Several prognostic scoring systems, including the Rockall score, Glasgow-Blatchford score (GBS), and Age, Blood tests, and Comorbidities (ABC) score, are routinely employed in clinical practice to predict outcomes in UGIB [3–5]. These scores incorporate variables such as age, hemodynamic status, laboratory findings, and endoscopic features, demonstrating reasonable prognostic performance. However, most existing scoring systems primarily reflect acute bleeding severity and physiological instability, often neglecting nutritional status and physiological reserve. This omission may reduce prognostic accuracy, particularly in frail patient populations.

Recently, the significance of nutritional status in influencing clinical outcomes and mortality in acute illnesses has garnered increasing attention. The prognostic nutritional index (PNI), first introduced by Onodera et al. [6], has been widely applied in gastrointestinal and oncological diseases [7, 8]. PNI is calculated from serum albumin levels and peripheral lymphocyte counts, reflecting both nutritional and immunological reserves. Albumin, a negative acute-phase reactant, declines rapidly in inflammatory states, while lymphocytopenia is associated with physiological stress and immune dysfunction [9]. Consequently, a low PNI may indicate severe inflammation, chronic malnutrition, or both. Although low PNI has been correlated with increased mortality and adverse outcomes in various clinical contexts, its prognostic value in patients with acute UGIB remains underexplored. Furthermore, the potential incremental benefit of combining PNI with established UGIB risk scores has yet to be clearly defined.

Lactate levels are frequently utilized as biochemical markers of acute bleeding and tissue hypoperfusion and have been associated with mortality in various disease states [10–12]. Lactate is produced during anaerobic metabolism and tends to accumulate under hypoxic conditions [13]. Measuring lactate levels is simple, cost-effective, and readily accessible. However, while lactate primarily indicates acute hemodynamic deterioration, it provides limited insight into the patient’s nutritional status and inflammatory reserve. This suggests that lactate and PNI may represent different yet complementary pathophysiological processes.

This study aims to evaluate the prognostic significance of PNI for 30-day mortality among patients admitted to the ED with acute UGIB and to determine whether incorporating PNI into established risk markers, such as the ABC score, Rockall score, and lactate level, enhances mortality prediction.

## Materials and methods

### Design and population

**Figure 1.**
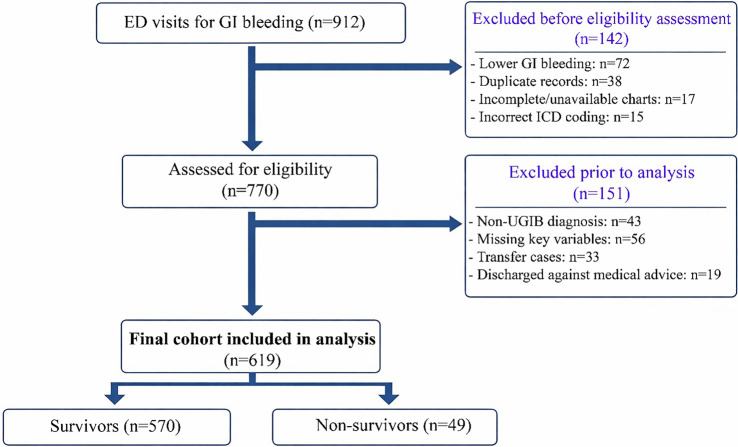
**Study flow diagram.** Of the 912 ED visits screened, 142 were excluded. Among the 770 encounters assessed, 151 were excluded, including 56 patients with missing key variables (19 with missing lactate levels and 35 with missing albumin levels). The final cohort comprised 619 patients, of whom 570 were survivors and 49 were non-survivors. Abbreviations: ED: Emergency department; UGIB: Upper gastrointestinal bleeding.

This retrospective study was conducted at the ED of Çanakkale Onsekiz Mart University Hospital from January 1, 2018, to December 31, 2024. The study population included 619 patients aged 18 years or older who presented to the ED with symptoms of acute UGIB and whose diagnosis was confirmed via endoscopy or clinically. For patients who did not undergo endoscopy, acute UGIB was clinically confirmed based on documented evidence of witnessed hematemesis, coffee-ground emesis, or melena, typically supported by an acute drop in hemoglobin and/or an elevated blood urea nitrogen level, after ruling out lower gastrointestinal sources. A total of 912 ED visits coded as gastrointestinal bleeding were screened. Prior to eligibility assessment, 142 encounters were excluded (lower GI bleeding, *n* ═ 72; duplicate records, *n* ═ 38; incomplete/unavailable charts, *n* ═ 17; incorrect ICD coding, *n* ═ 15). Among 770 encounters assessed for eligibility, 151 were excluded before analysis (non-UGIB diagnosis after chart review, *n* ═ 43; missing key variables required for risk score calculation, *n* ═ 56; inter-hospital transfer, *n* ═ 33; discharge against medical advice, *n* ═ 19). The final analytic cohort comprised 619 patients (Figure 1).

**Figure 2.**
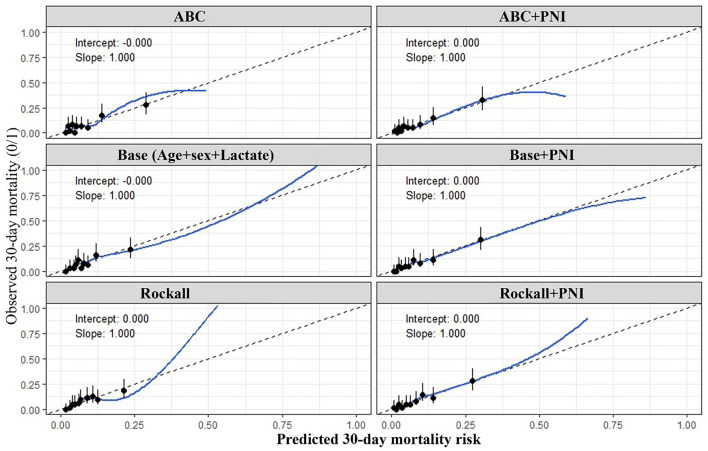
**Calibration plots for apparent prediction of 30-day mortality in patients with acute UGIB across the evaluated models.** Panels depict the agreement between predicted and observed 30-day mortality risk for the ABC score, ABC+PNI, the base model (age, sex, and lactate), Base+PNI, the pre-endoscopic Rockall score, and Rockall+PNI. The *x*-axis shows predicted 30-day mortality risk, and the *y*-axis shows observed 30-day mortality. The dashed 45∘ line represents perfect calibration. Black points with vertical error bars indicate decile-based observed event rates with corresponding 95% confidence intervals, and the blue line represents the LOESS-smoothed calibration curve across the risk spectrum. Calibration intercepts and slopes are displayed within each panel and reflect apparent model calibration. Abbreviations: UGIB: Upper gastrointestinal bleeding; ABC: Age, blood tests, and comorbidities; PNI: Prognostic nutritional index; LOESS: Locally estimated scatterplot smoothing; CI: Confidence interval.

### Data collection and definitions

Patients’ demographic characteristics, vital signs at admission, comorbidities, and medication history were retrospectively extracted from the hospital electronic information system. Laboratory parameters, including hemogram, biochemical markers, serum albumin, and lactate levels, were retrieved from medical records. Endoscopic findings and blood transfusion requirements were also documented. After applying the prespecified exclusions outlined in Figure 1, the final analytic cohort had complete data for all variables used in the primary regression and receiver operating characteristic (ROC) analyses, eliminating the need for data imputation or additional case-wise exclusions.

The primary variable of interest, the PNI, was calculated from admission laboratory values using the following formula:

*PNI ═ (10 × Serum Albumin, g/dL) + (0.005 × Total Lymphocyte Count, per mm^3^)*

To compare prognostic performance, established clinical risk scores were calculated for each patient in addition to the PNI. These included the pre-endoscopic Rockall score (clinical Rockall score), calculated using pre-endoscopic variables (age, hemodynamic status/shock, and comorbidity), excluding endoscopic diagnosis and stigmata of recent hemorrhage from the Rockall score calculation for this study; the GBS, which utilizes blood urea, hemoglobin, systolic blood pressure, and other clinical markers; and the ABC score (age, blood tests, and comorbidities), calculated according to the original validated definition. The ABC score assigns points for age 60–74 years (1) and ≥75 years (2); urea >10 mmol/L (1); albumin <3.0 g/dL (2); creatinine 100–150 µmol/L (1) and >150 µmol/L (2); comorbidity components including altered mental status (2), liver cirrhosis (2), disseminated malignancy (4), and American Society of Anesthesiologists (ASA) class 3 (1) or ≥4 (3). Total scores range from 0 to 18.

### Outcome measures

The primary outcome of this study was defined as all-cause 30-day mortality from the date of the index ED visit. Mortality status was verified by reviewing hospital electronic records and the national death notification system.

### Statistical analysis

The Kolmogorov–Smirnov test was utilized to assess the normality of continuous variables. Continuous variables were reported as medians with interquartile ranges (IQR), while categorical variables were presented as frequencies and percentages. Comparisons between survivor and non-survivor groups were performed using the Mann–Whitney *U* test for continuous variables. Categorical variables were analyzed using the Pearson chi-square test or Fisher’s exact test, as appropriate.

Univariable logistic regression models were constructed to report crude, unadjusted associations with 30-day mortality and were not used for variable selection. For multivariable modeling, we utilized a prespecified, clinically informed candidate set consisting of hematemesis, lactate, ABC score, and PNI. A forced-entry multivariable logistic regression model was applied to this prespecified set simultaneously. To avoid redundancy and potential collinearity, age and sex were not entered separately into models that already included the ABC score (which incorporates demographic/comorbidity components). Multicollinearity was assessed using variance inflation factors (VIF), with VIF >5 considered indicative of problematic collinearity. PNI was then included as the primary variable of interest to evaluate its adjusted association and incremental prognostic value beyond the prespecified model. The Hosmer and Lemeshow test was computed to assess goodness of fit in the multivariable models; a nonsignificant *P* value indicated a good fit. Model performance was evaluated using discrimination (AUROC), calibration (calibration intercept and slope, representing apparent performance), overall accuracy (Brier score), and visual calibration plots (Figure 2). Calibration plots were created by plotting observed vs predicted 30-day mortality rates, utilizing a locally estimated scatterplot smoothing (LOESS) curve and decile-based grouping for reference. This approach facilitates a visual assessment of agreement across the risk spectrum.

Commonly accepted high-risk thresholds from prior studies and clinical practice (ABC >5, pre-endoscopic Rockall ≥5, and lactate ≥2 mmol/L) were employed to enhance interpretability. Since no established cutoff exists for PNI in acute UGIB, the threshold (39.8) was derived post hoc from our cohort using ROC analysis (Youden index) to optimize discrimination for 30-day mortality. A covariate-adjusted ROC curve analysis was conducted using multivariable logistic regression to determine diagnostic performance metrics and to estimate the AUROC. Pairwise comparisons of correlated AUROCs were performed in R (version 3.6.2) using DeLong’s test for paired ROC curves (pROC package, roc.test(), method = “delong”) [14]. To control for multiple testing and strictly manage the Type I error rate, *P* values from these pairwise comparisons were adjusted using the Holm-Bonferroni method. For each comparison, ΔAUC, SE, 95% CI, Z statistic, and *P* value were extracted directly from the R output. All statistical analyses were conducted using SPSS 20.0 for Windows (IBM Corp., Armonk, NY, USA) and R software version 3.6.2. A two-sided *P* value < 0.05 was established as the threshold for statistical significance. These cutoff-based subgroup and combination analyses were performed for clinical interpretability and should be considered exploratory; all primary regression and ROC models treated predictors as continuous variables.

### Ethical statement

The study protocol received approval from the Clinical Research Ethics Committee (Decision No: 2025-130; April 17, 2025) and was conducted in accordance with the principles outlined in the Declaration of Helsinki. Given the retrospective design of the study, the requirement for informed consent was waived.

## Results

The patient selection process and exclusions are summarized in Figure 1. The study comprised 619 patients with acute UGIB, exhibiting an overall 30-day mortality rate of 7.9% (49/619). A comparison of baseline clinical characteristics between included (*n* ═ 619) and excluded (*n* ═ 56) patients revealed no significant systematic differences in major severity markers, thereby minimizing the risk of substantial selection bias (Table S1). Baseline demographic, clinical, and laboratory characteristics are detailed in Table 1. Patients in the mortality group were slightly older than survivors (median age: 74.0 [67.0–86.0] vs. 73.5 [63.0–81.0] years, *P* ═ 0.047), and exhibited significantly lower oxygen saturation upon admission. Hematemesis and the requirement for blood transfusions were more prevalent among patients who died. Endoscopy was performed in 481 out of 619 patients (77.7%). Endoscopic findings, as summarized in Table 1, were recorded from patients who underwent the procedure; multiple endoscopic findings per patient were permitted, with percentages presented using the total number of patients in each column as the denominator. Laboratory results indicated that serum albumin levels were significantly lower in the mortality group, while lactate levels were significantly higher. PNI values were significantly lower in deceased patients (37.6 [33.5–41.2] vs. 43.6 [38.8–49.1], *P* < 0.001). Furthermore, the ABC score, Rockall score, and GBS were all significantly elevated in the mortality group, as presented in Table 1.

**Table 1:** Demographics of patients presenting to the emergency department with acute upper gastrointestinal bleeding

**Parameters**	**All patient** ***n* ═ 619**	**Survivor** ***n* ═ 570**	**30-day mortality** ***n* ═ 49**	***P***
Age, years, med. (IQR)	74.0 (63.0--81.0)	73.5 (63.0–81.0)	74.0 (67.0–86.0)	0.047
Age Group, *n* (%)				
<60 years	127 (20.5)	122 (21.4)	5 (10.2)	0.155
≤60 -- > 80 years	296 (47.8)	271 (47.5)	25 (51.0)	
≥80 years	196 (31.7)	177 (31.1)	19 (38.8)	
Sex (M/F), *n* (%)	384 (62.0) / 235 (38.0)	350 (61.4) / 220 (38.6)	34 (69.4) / 15 (30.6)	0.269
Vital sign at admission, med. (IQR)				
SBP	120.0 (110.0–130.0)	120.0 (110.0–131.0)	119.0 (108.0–125.0)	0.151
DBP	74.5 (67.0–80.0)	75.0 (67.0–80.0)	70.0 (64.0–80.0)	0.093
Heart rate	88.0 (80.0–97.0)	87.5 (80.0–96.0)	90.0 (80.0–100.0)	0.143
Respiratory rate	16.0 (15.0–18.0)	16.0 (15.0–18.0)	16.0 (15.0–18.0)	0.806
Oxygen saturation	98.0 (97.0–99.0)	98.0 (97.0–99.0)	98.0 (95.0–98.0)	0.013
Chronic diseases, *n* (%)				
No	82 (13.2)	80 (14.0)	2 (4.1)	0.126
1	132 (21.3)	122 (21.4)	10 (20.4)	
2 or more	405 (65.4)	368 (64.6)	37 (75.5)	
Medication, *n* (%)				
None	83 (13.4)	79 (13.9)	4 (8.2)	0.185
Anti-platelet	187 (30.3)	176 (31.0)	11 (22.4)	0.212
Anti-coagulants	159 (25.8)	143 (25.2)	16 (32.7)	0.251
NSAIDs	86 (13.9)	80 (14.1)	6 (12.2)	0.721
Transfusion, *n* (%)	435 (70.4)	394 (69.2)	41 (83.7)	0.034
Endoscopy, *n* (%)	481 (77.7)	444 (77.9)	37 (75.5)	0.700
Endoscopic findings, *n* (%)				
Esophageal ulcer	49 (7.9)	44 (7.7)	5 (10.2)	0.536
Duodenal ulcer	53 (8.6)	50 (8.8)	3 (6.1)	0.525
Erosive gastritis	92 (14.9)	84 (14.7)	8 (16.3)	0.447
Gastric ulcer	163 (26.3)	154 (27.0)	9 (18.4)	0.187
Gastritis	262 (42.3)	239 (41.9)	23 (46.9)	0.496
Esophageal varices	30 (4.8)	24 (4.2)	6 (12.2)	0.025
Other	115 (18.6)	100 (17.5)	15 (30.6)	0.024
Laboratory				
Hemoglobin (g/dl)	8.8 (6.8–11.0)	8.8 (6.7–11.0)	9.1 (6.9–11.0)	0.592
Hematocrit (%)	27.0 (21.0–33.0)	27.0 (21.0–33.0)	27.6 (22.0–34.5)	0.468
Platelet (mm^3^ x 10^3^)	242.0 (180.5–311.0)	242.0 (181.0–306.0)	251.0 (178.0–383.0)	0.153
Urea (mg/dl)	63.0 (40.0–96.0)	62.0 (40.0–95.0)	72.0 (47.0–111.0)	0.071
ALT (U/l)	11.0 (8.0–17.0)	11.0 (8.0–16.0)	10.0 (6.0–18.0)	0.755
AST (U/l)	16.0 (12.0–22.0)	16.0 (12.0–22.0)	17.0 (12.0–23.0)	0.371
Albumin (g/dl)	3.5 (3.2–3.9)	3.6 (3.2–4.0)	3.3 (2.8–3.6)	<0.001
Lactate	1.7 (1.1–2.3)	1.6 (1.1–2.2)	2.3 (1.2–3.4)	0.001
Illness severity scores				
Pre-endoscopic Rockall score	5.0 (3.0–7.0)	5.0 (3.0–6.0)	6.0 (5.0–7.0)	<0.001
GBS score	11.0 (9.0–13.0)	11.0 (8.0–13.0)	13.0 (10.0–14.0)	0.031
ABC score	4.0 (2.0–6.0)	4.0 (2.0–6.0)	7.0 (4.0–10.0)	<0.001
PNI	43.2 (38.2–48.8)	43.6 (38.8–49.1)	37.6 (33.5–41.2)	<0.001

Endoscopic findings were documented in patients who underwent endoscopy (total *n* ═ 481). Multiple endoscopic findings per patient were permitted; thus, the categories are not mutually exclusive. The percentages of endoscopic findings are calculated using the total number of patients in each category as the denominator. Abbreviations: IQR: Interquartile range; med: Median; SBP: Systolic blood pressure; DBP: Diastolic blood pressure; NSAID: Nonsteroidal anti-inflammatory drugs; ALT: Alanine aminotransferase; AST: Aspartate aminotransferase; GBS: Glasgow-Blatchford score; ABC: Age, blood tests, and comorbidities; PNI: Prognostic nutritional index.

In univariable logistic regression analysis (LRA), oxygen saturation, hematemesis, transfusion requirement, serum albumin level, lactate level, Rockall score, GBS, ABC score, and PNI were significantly associated with 30-day mortality. In multivariable LRA, hematemesis (OR: 2.077 [1.048–4.115]), lactate level (OR: 1.225 [1.047–1.434]), ABC score (OR: 1.201 [1.053–1.370]), and PNI (OR: 0.847 [0.765–0.938]) emerged as independent predictors of 30-day mortality. No significant multicollinearity was detected across the evaluated models (all VIFs ranged from 1.01 to 1.21). The multivariable model demonstrated adequate goodness-of-fit according to the Hosmer–Lemeshow test (χ^2^ ═ 8.479, df = 8, *P* ═ 0.388). Each one-unit increase in PNI was associated with a 15.3% reduction in mortality risk. Results of regression analyses are summarized in Table 2.

**Table 2:** Univariable and multivariable logistic regression analyses identifying predictors of 30-day mortality

	**30-day mortality**			
	**Univariate** **Odds ratio (95% CI)**	***P***	**Multivariate** **Odds ratio (95% CI)**	***P***
Age years	1.029 (1.006–1.053)	0.053		
<60 (Reference)	-			
≤60 -- >80	2.251 (0.842–6.020)	0.106		
≥80	2.619 (0.952–7.204)	0.062		
Sex/Female (Reference)	1.425 (0.758–2.676)	0.271		
Oxygen saturation	0.879 (0.800–0.965)	0.007		
Hematemesis	1.728 (1.043–2.862)	0.034	2.077 (1.048–4.115)	0.036
Transfusion	2.454 (1.257–4.788)	0.009		
Endoscopic findings				
Esophageal varices	3.174 (1.231–8.183)	0.017		
Other endoscopic findings	2.074 (1.088–3.951)	0.027		
Laboratory				
Albumin	0.375 (0.233–0.602)	<0.001		
Lactate	1.403 (1.217–1.619)	<0.001	1.225 (1.047–1.434)	0.011
Illness severity scores				
Pre-endoscopic Rockall score	1.282 (1.118–1.470)	<0.001		
GBS	1.100 (1.009–1.199)	0.031		
ABC score	1.380 (1.251–1.521)	<0.001	1.201 (1.053–1.370)	0.006
PNI	0.888 (0.850–0.927)	<0.001	0.847 (0.765–0.938)	0.001

Abbreviations: CI: Confidence interval; GBS: Glasgow-Blatchford score; ABC: Age, blood tests, and comorbidities; PNI: Prognostic nutritional index.

When PNI was analyzed in conjunction with clinical risk scores, mortality risk was clearly differentiated. These cutoff-based combination analyses were conducted for interpretability and should be regarded as exploratory. Patients with an ABC score ≤5 and PNI>39.8 experienced a mortality rate of 2.7%, while those with an ABC score >5 and PNI ≤39.8 had a mortality rate of 25.8%, representing a 12.5-fold higher mortality risk compared to the reference group (crude OR 12.50 [5.60–27.92]). Similarly, the combination of a pre-endoscopic Rockall score ≥5 and low PNI was associated with a 10.6-fold higher mortality risk (crude OR 10.58 [4.02–27.87]). Patients with lactate levels ≥2 mmol/L and low PNI had a mortality rate of 26.4%, corresponding to a 16.6-fold higher risk (crude OR 16.60 [6.07–45.40]). These combined analyses are presented in Table 3. To address the data-derived nature of the 39.8 cutoff, we re-evaluated these risk groups using a clinically practical threshold of PNI < 40. Patient classification and subsequent absolute mortality risks remained virtually unchanged, confirming the robustness of these exploratory findings.

**Table 3:** Association between clinical risk scores and mortality in patients with acute upper gastrointestinal bleeding

	**30-day mortality**		
	**Deaths/total, *n*/N (% [95% CI])**	**Crude model, OR (95% CI)**	***P* value**
***ABC score | PNI***			
ABC score ≤ 5 | PNI>39.8 (Reference)	9/333 (2.7% [1.3–5.1])	-	-
ABC score ≤ 5 | PNI≤39.8	10/112 (8.9% [4.8–15.8])	3.529 (1.396–8.924)	0.008
ABC score > 5 | PNI>39.8	5/77 (6.5% [2.8–14.5])	2.500 (0.814–7.682)	0.110
ABC score > 5 | PNI≤39.8	25/97 (25.8% [17.9–35.5])	12.500 (5.597–27.918)	<0.001
***Rockall score | PNI***			
Pre-endoscopic Rockall score < 5 | PNI>39.8 (Reference)	5/210 (2.3% [1.0–5.5])	-	-
Pre-endoscopic Rockall score < 5 | PNI≤39.8	4/54 (7.4% [2.9–17.6])	3.280 (0.850–12.660)	0.085
Pre-endoscopic Rockall score ≥ 5 | PNI>39.8	8/199 (4.0 % [2.0–7.7])	1.717 (0.552–5.341)	0.350
Pre-endoscopic Rockall score ≥ 5 | PNI≤39.8	32/156 (20.5% [14.8–27.6])	10.581 (4.017–27.870)	<0.001
***Lactate | PNI***			
Lactate<2 | PNI > 39.8 (Reference)	5/236 (2.1% [0.9–4.9])		
Lactate<2 | PNI ≤ 39.8	13/123 (10.5% [6.1 - 17.3])	5.460 (1.899–15.698)	0.002
Lactate ≥2 | PNI > 39.8	8/173 (4.6% [2.3–8.9])	2.240 (0.720–6.970)	0.164
Lactate ≥2 | PNI ≤ 39.8	23/87 (26.4% [18.2–36.7])	16.603 (6.072–45.402)	<0.001

Abbreviations: OR: Odds ratio; CI: Confidence interval; ABC: Age, blood tests, and comorbidities; PNI: Prognostic nutritional index.

**Table 4:** Impact of PNI, ABC score, and pre-endoscopic Rockall Score on the discrimination accuracy of mortality prediction models

	**Area under the ROC curve**		**Pairwise analysis**						
	**(95% CI)**		**(95% CI)**						
	**Without PNI**	**With PNI**	****Δ**AUC**	**SE**	**Lower**	**Upper**	**Z statistic**	***P***	**Adjusted *P***
Base model = age, sex and lactate	0.708 (0.636–0.781)	0.774 (0.707–0.840)	--0.065	0.033	--0.131	0.000	--1.968	0.049	0.098
ABC score	0.748 (0.672–0.824)	0.782 (0.711–0.852)	--0.033	0.015	--0.062	--0.004	--2.259	0.024	0.096
Base model + ABC score	0.764 (0.693–0.835)	0.791 (0.722–0.859)	--0.027	0.019	--0.065	0.011	--1.384	0.166	0.166
Pre-endoscopic Rockall score	0.681 (0.610–0.751)	0.765 (0.699–0.831)	--0.084	0.024	--0.131	--0.037	--3.528	<0.001	<0.001
Base model + pre-endoscopic Rockall score	0.729 (0.660–0.798)	0.782 (0.715–0.850)	--0.053	0.025	--0.102	--0.004	--2.129	0.033	0.099

A negative ΔAUC value signifies enhanced discriminative performance (AUROC) with the inclusion of PNI. Abbreviations: CI: Confidence interval; ABC: Age, blood tests, and comorbidities; PNI: Prognostic nutritional index; SE: Standard error; ΔAUC: Difference in area under the ROC curve (AUC [without PNI] - AUC [with PNI]); ROC: Receiver operating characteristic.

**Figure 3.**
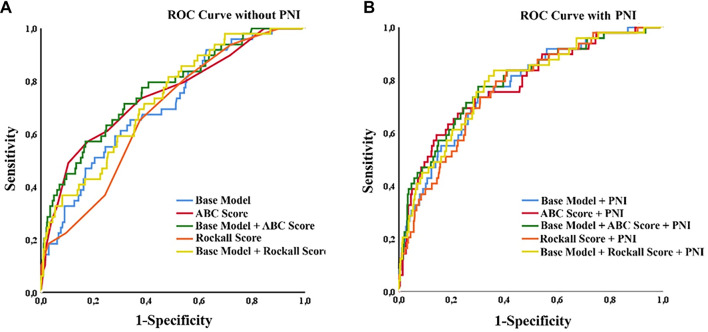
**Receiver operating characteristic curves for prediction of 30-day mortality in acute UGIB across models with and without PNI.** (A) Models without PNI: The base model (age, sex, and lactate), ABC score alone, base model + ABC score, pre-endoscopic Rockall score alone, and base model + pre-endoscopic Rockall score. (B) Corresponding models with PNI added: Base model + PNI, ABC score + PNI, base model + ABC score + PNI, pre-endoscopic Rockall score + PNI, and base model + pre-endoscopic Rockall score + PNI. The y-axis represents sensitivity, and the *x*-axis represents 1 - specificity. Across the evaluated models, the addition of PNI was associated with numerical improvement in discrimination, with statistically significant improvement in selected paired comparisons, as detailed in Table 4. Abbreviations: ROC: Receiver operating characteristic; UGIB: Upper gastrointestinal bleeding; PNI: Prognostic nutritional index; ABC: Age, blood tests, and comorbidities; AUROC: Area under the receiver operating characteristic curve.

ROC curve analysis indicated that the base model, which included age, sex, and lactate, had an AUROC value of 0.708. The addition of PNI to this model increased the AUROC to 0.774, a statistically significant difference (*P* ═ 0.049, adjusted *P* ═ 0.098). Incorporating PNI into the pre-endoscopic Rockall score raised the AUROC from 0.681–0.765 (*P <* 0.001, adjusted *P* < 0.001). While the addition of PNI to the ABC score resulted in a numerical AUROC increase (unadjusted *P* ═ 0.024), this difference was not significant after Holm-Bonferroni adjustment (adjusted *P* ═ 0.096). The increase observed in the Base Model + ABC score was also numerical and non-significant. Detailed AUROC values, 95% confidence intervals, and pairwise comparisons using the DeLong test are summarized in Table 4, with graphical representations presented in Figure 3. Across the evaluated models, calibration intercepts were approximately 0, and calibration slopes were approximately 1. Notably, the addition of PNI was associated with lower Brier scores, indicating improved overall predictive performance (Base model: 0.0679 vs. Base+PNI: 0.0638; ABC score: 0.0658 vs. ABC+PNI: 0.0646; pre-endoscopic Rockall score: 0.0686 vs. Rockall+PNI: 0.0658). Internal validation using stratified 5-fold cross-validation and bootstrap optimism correction confirmed that the discrimination gains associated with the addition of PNI were preserved. Additionally, decision curve analysis (DCA) demonstrated that incorporating PNI into clinical models provided an improved net clinical benefit across clinically relevant threshold probabilities (Tables S2 and S3).

## Discussion

This study identified PNI as an independent and robust predictor of 30-day mortality in patients presenting to the ED with acute UGIB. The overall mortality rate within the study population was 7.9%. In deceased patients, the PNI value was found to be 37.6 (33.5–41.2), significantly lower than that of survivors (43.6 [38.8–49.1], *P <* 0.001). In multivariable logistic regression, PNI remained independently associated with mortality when included alongside lactate levels and ABC scores. Our results indicate that each one-unit increase in PNI correlates with a 15% reduction in mortality risk (OR: 0.847; 95% CI: 0.765–0.938). Collectively, these findings underscore the significant role of a patient’s nutritional and immune reserve on clinical outcomes in conditions of high physiological stress, such as acute UGIB.

The Rockall and GBSs are established scoring systems utilized for risk assessment in acute UGIB [15–17]. In existing literature, these scores have demonstrated moderate discriminative ability for predicting mortality [18]. For instance, a recent study by Frías-Ordoñez et al. [19] validated the Rockall score, reporting moderate-to-good discrimination for mortality outcomes; the pre-endoscopy Rockall score achieved an AUC of 0.756 for one-month mortality and 0.762 for three-month mortality, indicating a “moderate” prognostic performance rather than exceptional accuracy. Likewise, Nik Pa et al. [20] reported that the discriminative performance of common ED scores for in-hospital mortality was moderate overall, with AUC values ranging from 0.638 (GBS) to 0.667 (pre-Rockall), reflecting only modest-to-moderate separation between survivors and non-survivors. In our study, both Rockall and GBS scores were significantly higher in the mortality group compared to survivors (6.0 [5.0–7.0] vs. 5.0 [3.0–6.0] and 13.0 [10.0–14.0] vs. 11.0 [8.0–13.0], respectively). However, in ROC analyses, while the AUROC value of the pre-endoscopic Rockall score alone was 0.681, the inclusion of PNI raised this value to 0.765, suggesting that nutritional status provides an important complementary contribution to traditional clinical scoring systems. When PNI was added to ABC-based models, discrimination improved numerically, achieving statistical significance in select comparisons (Table 4).

In recent years, the prognostic role of lactate levels in acute UGIB has garnered significant attention [21–23]. Studies have demonstrated that elevated serum lactate levels are associated with adverse outcomes, including increased mortality, prolonged hospitalization, and the necessity for intensive care unit (ICU) admission [21, 24]. However, the utility of lactate levels in predicting outcomes for UGIB remains contentious; some research indicates no significant correlation, while others find that elevated lactate levels are linked to poorer patient outcomes [23, 24]. Given these conflicting results, further investigation into the prognostic value of lactate across diverse patient populations is essential. In our cohort, lactate concentrations were significantly higher in the mortality group compared to survivors (2.3 [1.2–3.4] vs. 1.6 [1.1–2.2] mmol/L, *P* ═ 0.001). Multivariable analyses identified lactate level as an independent predictor of mortality (OR: 1.225; *P* ═ 0.011).

The prognostic significance of PNI has been established in various clinical contexts, including oncology, cardiovascular diseases, cirrhosis, and intensive care populations [25–30]. Particularly among critically ill patients, low PNI has been correlated with increased mortality, higher infection rates, and extended hospitalization [31, 32]. However, research regarding the application of PNI in patients with acute UGIB is limited. The existing literature includes a study by Li et al. [33], which assessed PNI in children with Henoch–Schönlein purpura and UGIB, indicating that low PNI proved useful for predicting the development of duodenal ulcers and hospitalization risk, but not mortality outcomes. In contrast, our study contributes to the existing body of knowledge by demonstrating the prognostic performance of PNI for mortality prediction in patients with acute UGIB, underscoring its clinically relevant application. Moreover, our findings suggest that PNI not only correlates with mortality but also significantly enhances the prognostic accuracy of established risk scores.

While PNI has traditionally been regarded as a marker of nutritional status, serum albumin, which functions as a negative acute-phase reactant, may decrease in various disease states, including systemic inflammation and acute physiological stress. Thus, in the context of acute UGIB, PNI likely reflects a combination of nutritional status, inflammatory burden, and acute disease severity [9]. This distinction is critical to prevent overstating the “nutritional” aspect of PNI in an ED setting. Furthermore, PNI is calculated using laboratory parameters routinely collected at ED admission and does not necessitate additional testing [6]. Consequently, it can be seamlessly integrated into the ED workflow. However, due to its overlap with existing risk models incorporating albumin or other disease severity markers, PNI should be viewed not as a replacement for established scores but as a complementary tool that may enhance early risk stratification to a limited extent. Thus, any incremental benefits should be interpreted cautiously and not as entirely independent signals.

A key finding of this study pertains to the combined use of PNI with clinical risk scores and lactate levels in assessing mortality risk. When PNI was evaluated alongside these parameters, the risk of mortality increased substantially. For instance, patients with an ABC score >5 and a PNI ≤39.8 exhibited a mortality rate of 25.8%, approximately 10.6 times higher than the reference group. A similar trend was noted for other combinations, with mortality risk increasing roughly 10.6-fold in patients with a pre-endoscopic Rockall score ≥5 and low PNI, and 16.6-fold in those with lactate levels ≥2 mmol/L and low PNI.

These results indicate that reliance on a single score may be insufficient in clinical practice, and that a multidimensional risk assessment can yield more reliable information. This interpretation is further supported by ROC analysis, which demonstrated that the AUROC of the base model (age, sex, and lactate) was 0.708, increasing to 0.774 after incorporating PNI, a difference that approached statistical significance (*P* ═ 0.049). These findings suggest that PNI may serve as a complementary parameter, associated with a modest enhancement in discriminative ability.

Several limitations merit consideration. First, the retrospective, single-center design may limit the generalizability of our findings to broader populations and healthcare settings. Second, PNI was assessed based on a single measurement at ED admission. Given that nutritional status and inflammatory response are dynamic processes, the absence of serial measurements may have constrained a comprehensive evaluation of its prognostic impact. Third, variations in treatment strategies for acute UGIB were not thoroughly analyzed, potentially introducing confounding factors affecting mortality. Fourth, albumin and lymphocyte count, the primary components of PNI, reflect not only nutritional status but also inflammation and acute stress response; thus, the underlying mechanisms could not be distinctly separated. Additionally, the PNI cutoff was derived from our cohort using ROC analysis and requires external validation for broader applicability. Fifth, missing data were addressed through a complete-case analysis approach, which presents a potential risk of selection bias. While our discrimination metrics underwent optimism correction via internal validation, the reported calibration parameters reflect apparent performance. Finally, only 30-day mortality was evaluated, while other clinical outcomes were not assessed.

## Conclusion

In conclusion, this study establishes that PNI is an independent predictor of 30-day mortality in patients presenting to the ED with acute UGIB. Low PNI values, particularly when combined with elevated clinical risk scores or lactate levels, are associated with a substantially increased risk of mortality, reaching up to 16.6 times in high-risk groups. Furthermore, the addition of PNI to existing clinical models and risk scores resulted in meaningful improvements in discriminative ability, statistically significant for specific clinical scores such as the pre-endoscopic Rockall score. Given that PNI is a simple, low-cost, and widely available index, it may enhance early prognostic stratification and therapeutic planning in acute UGIB, supporting a more integrated prognostic approach.

## Supplemental data

Supplemental data are available at the following link: https://www.bjbms.org/ojs/index.php/bjbms/article/view/13995/4147.

## Data Availability

The data supporting the findings of this study are available from the corresponding author upon reasonable request and can be provided for peer review if necessary.
